# High Photocatalytic Activity of g-C_3_N_4_/La-N-TiO_2_ Composite with Nanoscale Heterojunctions for Degradation of Ciprofloxacin

**DOI:** 10.3390/ijerph19084793

**Published:** 2022-04-15

**Authors:** Yanmin Yu, Ke Liu, Yangyang Zhang, Xuan Xing, Hua Li

**Affiliations:** 1 School of Basic Medicine, Hubei University of Arts and Science, Xiangyang 441053, China; yuyanmin2005@163.com (Y.Y.); yangyangzhang2017@163.com (Y.Z.); 2 Hubei Key Laboratory of Low Dimensional Optoelectronic Materials and Devices, Hubei University of Arts and Science, Xiangyang 441053, China; liuke@hbuas.edu.cn; 3 College of Life and Environmental Sciences, Minzu University of China, Beijing 100081, China

**Keywords:** photocatalysis, ciprofloxacin, g-C_3_N_4_, TiO_2_, composite material, wastewater, organic micropollutants

## Abstract

Ciprofloxacin (CIP) in natural waters has been taken as a serious pollutant because of its hazardous biological and ecotoxicological effects. Here, a 3D nanocomposite photocatalyst g-C_3_N_4_/La-N-TiO_2_ (CN/La-N-TiO_2_) was successfully synthesized by a simple and reproducible in-situ synthetic method. The obtained composite was characterized by XRD, SEM, BET, TEM, mapping, IR, and UV-vis spectra. The photocatalytic degradation of ciprofloxacin was investigated by using CN/La-N-TiO_2_ nanocomposite. The main influential factors such as pH of the solution, initial CIP concentration, catalyst dosage, and coexisting ions were investigated in detail. The fastest degradation of CIP occurred at a pH of about 6.5, and CIP (5 mg/L starting concentration) was completely degraded in about 60 min after exposure to the simulated solar light. The removal rates were rarely affected by Na^+^ (10 mg·L^−1^), Ca^2+^ (10 mg·L^−1^), Mg^2+^ (10 mg·L^−1^), and urea (5 mg·L^−1^), but decreased in the presence of NO_3_^−^ (10 mg·L^−1^). The findings indicate that CN/La-N-TiO_2_ nanocomposite is a green and promising photocatalyst for large-scale applications and would be a candidate for the removal of the emerging antibiotics present in the water environment.

## 1. Introduction

Antibiotic pollution poses a serious threat to human health through drinking water and/or the food-chain, especially in developing countries, and how to efficiently remove it has gained great attention over the past [1,2,3,4,5,6]. Fluoroquinolones (FQs) are one of the most consumed antibiotics and have a total consumption of about 25 million kilograms every year in China [7,8]. Ciprofloxacin (CIP), as the second-generation fluoroquinolone, is extensively used in humans and animals to sterilize and fight infections due to their good oral intake and large activity spectrum [9]. Ultimately, it enters the WWTPs (wastewater treatment plants) as a result of being poorly adsorbed in the body and excreted unchanged in feces and urine [10,11]. As the quinolone ring is degraded difficultly, CIP is inefficiently removed by most wastewater and sewage treatment plants via conventional wastewater treatment technologies or is directly poured into water bodies in countries lacking effluent treatments [12]. CIP has been detected frequently in various groundwater [13], surface water [14], and seawater [15] throughout the world [12,13,14,15,16,17,18,19,20]. Therefore, an effective treatment technology is necessary to transform CIP into noN-Toxic, pharmaceutically less active, or more biodegradable species in order to face CIP pollution problems in water.

Semiconductor photocatalysis, which has attracted wide attention by virtue of low- cost, energy-savings, non-toxicity, and high catalytic degradation activity of organic pollutants in purifying water under solar-light irradiation, has been employed to address the problem of antibiotic wastewater and it has made great progress over the past decade [19,20,21,22,23]. A series of previous studies was focused on the photocatalytic degradation of CIP and the data are listed in Table 1. Gad-Allah et al. employed commercial anatase TiO_2_ to degrade CIP under simulated sunlight, and the best reaction rate was obtained in natural ciprofloxacin pH (5.8) and 1000 mg/L TiO_2_ [24]. Wen et al. synthesized a novel Z-scheme CeO_2_–Ag/AgBr photocatalyst for photocatalytic degradation of ciprofloxacin, the degradation rate could reach 93.05% in 120 min under visible light irradiation [25]. Rahmani-Aliabadi et al. evaluated the photocatalytic activities of FeS/Fe_2_S_3_/zeolite by photodegradation of ciprofloxacin, and the best catalysis conditions are pH 3.7, catalyst dose of 2.8 g·L^−1^, 3.8 mg·L^−1^ of CIP for 102 min irradiation of the suspension [26]. Gan et al. employed different post−treatment methods to synthesize three kinds of mesoporous TiO_2_ nanocrystallite aggregates and comparatively studied the removal capability of CIP by adsorption in the dark and photocatalysis under light irradiation [27]. El-Kemary et al. carried out systematic research on the photocatalytic activity of the prepared ZnO nanoparticles for the degradation of ciprofloxacin drug under UV light irradiation in aqueous solutions of different pH values [28]. Li et al. successfully prepared the magnetic 3D γ-Fe_2_O_3_@ZnO core-shell photocatalyst, which exhibits excellent degradation efficiency of CIP up to 92.5% under simulated sunlight irradiation [29]. Chen et al. studied the CIP decay over Bi_2_O_3_/(BiO)_2_CO_3_ heterojunctions under simulated solar light irradiation, where the removal efficiency achieved 93.4% in 30 min [30], and so on [16,20,29,31,32,33,34,35,36,37,38,39,40,41].

From Table 1, it is can be found that the existing research mainly focuses on simulating wastewater degradation in the laboratory and little research on the catalytic degradation of CIP by the semiconductor photocatalytic systems in different water matrices (river water, synthetic wastewater similar by composition to wastewater of pharmaceutical industry). Therefore, further optimization and innovation of the semiconductor photocatalysts for catalytic degradation of CIP in actual wastewater are still in great demand. Hence, in this work, the semiconductor photocatalysis (CN/La-N-TiO_2_) will be applied to remove ciprofloxacin in an actual water environment.

TiO_2_-assisted photocatalysis has attracted widespread attention to purify polluted water by the removal of organic pollutants, owing to its good stability, low-budget, non-toxicity, and excellent photocatalytic performance [42,43]. It has been demonstrated that TiO_2_ doped with elements such as N, P, S, B, C, Co, and La, is an effective way to introduce impurity energy levels, change the intrinsic band structure, reduce the bandgap and improve the photocatalytic capability under visible light [39,41,44]. Lanthanide is a good candidate because its low-valent doping results in the formation of oxygen vacancies on the exterior or surface lattice and hence improves the catalytic activity [41,45,46]. In our previous research work, TiO_2_ co-doped with nitrogen and lanthanum/gadolinium ([Xe]5d^1^6s^2^/[Xe]4f^7^5d^1^6s^2^ electronic configuration) was successfully synthesized using urea and lanthanum nitrate as a precursor, and the result is that La or Gd-N codoping could significantly improve the photocatalytic activity and thermal stability of TiO_2_ [42,47]. In addition to ion doping, the highly optically active heterojunction structures also could effectively reduce recombination of electron-hole pairs, broaden the spectral response range and enhance photocatalytic activity. g-C_3_N_4_ nanosheets with hybrid engineering architectures exhibit superior chemical stability and photocatalytic decomposition activity of organic pollutants under visible light due to distinct electronics and morphological behavior [22,23,34,41,48,49]. The addition of g-C_3_N_4_ nanosheet with La-N-TiO_2_, as the appropriate band position of La-N-TiO_2_ can match well with that of g-C_3_N_4_ to effectively hinder the recombination of photo-generated carriers, which improves the intrinsic electrical conductivity, results in a good electrochemical performance of g-C_3_N_4_/La-N-TiO_2_ composites. 

Hence, in this work, the composite materials, made of graphitic carbon nitride/lanthanum and nitrogen co-doped titanium dioxide (g-C_3_N_4_/La-N-TiO_2_), have been successfully fabricated for the efficient photocatalytic degradation of ciprofloxacin (CIP). A 96.8% degradation of CIP was achieved in 60 min under the conditions of 0.75 g·L^−1^ CN/La-N-TiO_2_, 10 mg·L^−1^ CIP, and pH of 6.5. It was applied to remove CIP in the collected Danjiangkou Water sample, and the degradation efficiency was close to that of laboratory simulated wastewater. The intermediates and the suggested mineralization pathway of CIP were analyzed and discussed. The findings indicate that CN/La-N-TiO_2_ nanocomposite is a green and promising photocatalyst for large-scale applications and would be a candidate for the removal of the emerging antibiotics present in the water environment.

## 2. Materials and Methods

### 2.1. Materials

We purchased urea (CH_4_N_2_O), La(NO_3_)_3_, dicyandiamideand (99%), and TiCl_4_ from Sinopharm Chemical Reagent Co. (Shanghai, China) Ti(OCH(CH_3_)_2_)_4_ and P123 (PEO-PPO-PEO) were purchased by Sigma-Aldrich. The water used throughout all of the experiments was purified using a Millipore system.

### 2.2. Synthesis of g-C_3_N_4_/La-N-TiO_2_ (CN/La-N-TiO_2_) Composite Nanomaterials

g-C_3_N_4_ was prepared according to a reported procedure [50]. Given amounts of P123, La(NO_3_)_3_, and urea were mixed in 20 mL anhydrous ethanol, 0.44 mL TiCl_4_, and 2.1 mL (CH_3_CH_3_CHO)_4_Ti were added after vigorous stirring for 2 h at room temperature, then it continued to stir for 3 h. The resulting mixture was sealed in a crucible with a cover to aging for 48 h at room temperature. Before drying at 60 °C for 8 h, a certain amount of dicyandiamide (20, 30, 50, 70, or 100 mg) was added. Then, the dried samples were subjected to calcination at the temperature of 580 °C for 5 h with a heating rate of 2.0 °C·min^−1^. Finally, g-C_3_N_4_/La-N-TiO_2_(CN/La-N-TiO_2_) powder products were obtained and ground thoroughly. In control experiments, the samples of different elements doped were synthesized under identical conditions, marked as La-N-TiO_2_, CN/N-TiO_2_, and CN/La-TiO_2_.

### 2.3. Spectroscopic and Electron Microscopic Characterization

Physicochemical properties of the prepared catalyst were thoroughly characterized as follows: X-ray diffraction (XRD) patterns were recorded on XD-3 X-ray diffraction, fitted with a Cu Kα radiation (λ = 1.54 Å). Fourier transform infrared (-FTIR) spectroscopy was measured on a Bruker VERTEX 70v FTIR Spectrometer for samples embedded in KBr pellets. Surface morphologies and structure features were observed with a field emission scanning electron microscope (FE-SEM, Hitachi s-4800, Tokyo, Japan) at an accelerating voltage of 10 kV, transmission electron microscopic (TEM, FEI-Talos F200S, Hong Kong, China) at an accelerating voltage of 200 kV, and STEM mapping. UV-vis diffuse reflectance spectra (DRS) of the samples were collected on a Perkin Elmer Lambda 950 UV-vis spectrophotometer equipped with an integrating sphere attachment (BaSO_4_ as the reference) within the range of 200–1000 nm. The elemental composition and the chemical state of the samples were confirmed by X-ray photoelectron spectroscopy (Thermo Scientific Escalab 250Xi, Waltham, MA, USA) with a Mg Kα source.

### 2.4. Photocatalytic Tests

The visible light photocatalytic performances of CN/La-N-TiO_2_, CN/N-TiO_2_, CN/La-TiO_2_, and La-N-TiO_2_ were evaluated by the degradation of ciprofloxacin in an aqueous solution containing a concentration of (5–20) mg/L ciprofloxacin and 0.75 g/L catalyst in 200 mL glass vessels. The suspension was stirred vigorously in dark for 30 min to achieve adsorption-desorption equilibrium between the catalysts and the ciprofloxacin, and then was irradiated under a 350 W Xe lamp light source (PLS-SXE300, Shanghai Bilang Plant, Shanghai, China) with an optical filter, λ > 420 nm. Sample was taken out periodically of the reactor, then centrifuged at 9000 rpm and analyzed by a UV-vis spectrophotometer (JASCO V-750, Tokyo, Japan). The rate of photocatalytic ciprofloxacin degradations was obtained by recording the changes in absorbance of the reaction solution. HR-MS (Thermo Scientific Q Exactive, Waltham, MA, USA) was used to analyze the byproducts formed during the photocatalytic degradation of CIP.

## 3. Results and Discussion

### 3.1. Photocatalysts Characterization

#### 3.1.1. XRD and -FTIR Spectra

**Figure 1:**
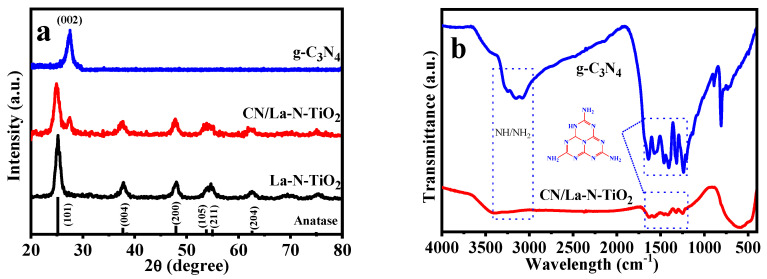
XRD patterns (**a**) of g-C_3_N_4_, La-N-TiO_2_, and CN/La-N-TiO_2_, and FTIR spectra (**b**) of La-N-TiO_2_ and CN/La-N-TiO_2_.

The crystal structure of the as-prepared g-C_3_N_4_, La-N-TiO_2_, and CN/La-N-TiO_2_ was characterized by XRD, and the results are shown in Figure 1a. The diffraction peaks at 2θ = 25.29°, 37.87°, 48.09°, 53.83°, 55.13°, and 62.81° in the XRD pattern of La-N-TiO_2_/CN/La-N-TiO_2_ correspond to (101), (004), (200), (105), (211), and (204) diffraction planes of anatase TiO_2_ (JCPDS No. 98-4921), correspondingly [34]. The diffraction peak at 2θ = 27.0° (indexed as the (002) peak for graphitic materials) of g-C_3_N_4_ is attributed to the interplanar stacking of aromatic systems, which slightly shifted to 27.61° following the hybridization of La-N-TiO_2_ and g-C_3_N_4_, revealing the reduction of layer spacing and the formation of denser g-C_3_N_4_ structures [23,51]. It is obvious that La-N-TiO_2_ and CN/La-N-TiO_2_ exhibit similar diffraction patterns, except for a characteristic peak at 2θ = 27.0° for CN/La-N-TiO_2_, so it can be concluded that g-C_3_N_4_ was successfully decorated in the composite. The amount of dicyandiamide on the crystal structure of CN/La-N-TiO_2_ composite was investigated (Figure S1a, Supporting Information). In Figure S1b, when the amount of dicyanide added is 70 mg, CN/La-N-TiO_2_ composite exhibits the strongest photocatalytic activity. In this study, the optimal amount of dicyanide is 70 mg.

FTIR was used to further study the surface functional groups and chemical structures of g-C_3_N_4_ and CN/La-N-TiO_2_ (Figure 1b). The vibration peaks in the range of 1200–1700 cm^−1^ are attributed to the typical CN heterocycles rings stretching [52]. The characteristic absorption band at 811 cm^−1^ could be observed, which is commonly ascribed to an s-triazine ring system [53]. The broad band adsorption peak in the range of 3150–3340 cm^−1^ corresponds to the stretching vibration modes of the terminal NH_x_ group [54,55]. In the case of CN/La-N-TiO_2_, in addition to the above peaks of g-C_3_N_4_, there are other strong absorption peaks at 500–800 cm^−1^, which are related with the Ti-O and Ti-O-Ti bands [34]. 

#### 3.1.2. Morphology Analysis

**Figure 2:**
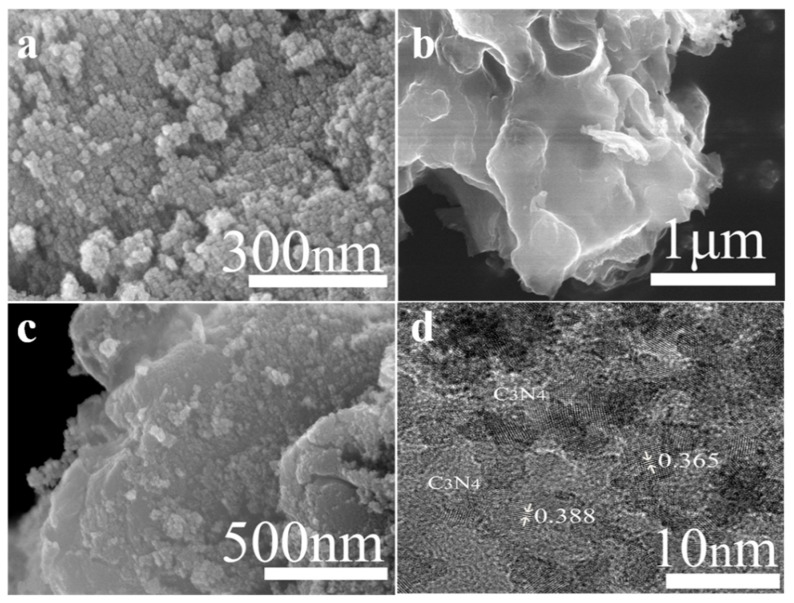
SEM images of La-N-TiO_2_ (**a**), g-C_3_N_4_ (**b**), CN/La-N-TiO_2_ (**c**), and TEM images of CN/La-N-TiO_2_ (**d**).

The surface morphology and structure of representative samples were examined by SEM and TEM (Figure 2). It can be found from Figure 2a that the appearance of these incompletely separated La-N-TiO_2_ nanoparticles is spherical-like -nanoparticles with an average particle size of 10–20 nm. Figure 2b shows that the 2D-g-C_3_N_4_ nanosheets own curved nanosheets with lamellar morphology, and their surface is smooth and soft. As Figure 2c shows, the surface of the g-C_3_N_4_ nanosheet is covered by the La-N-TiO_2_ particles to form a highly uniform composite heterojunction structure of the CN/La-N-TiO_2_ composite. The decorated and robustly 2D-g-C_3_N_4_ attached with the structure of the La-N-TiO_2_ nanoparticle to form a 3D nanocomposite photocatalyst g-C_3_N_4_/La-N-TiO_2_(CN/La-N-TiO_2_), which can provide a larger surface area, enhance the surface permeability and porous property. The TEM image provides a more evident observation of the two components in Figure 2d. The black and dark gray irregular dots located on the g-C_3_N_4_ nanosheet were from La-N-TiO_2_ nanoparticles. The darker part with a spherical shape should be La-N-TiO_2_ and the lighter part is g-C_3_N_4_. The size of La-N-TiO_2_ in the CN/La-N-TiO_2_ composite is smaller than that of the pristine La-N-TiO_2_, which is due to the confinement of CN against the self-assembly of small component TiO_2_ nanoparticles to a certain extent [56]. Two clearly crystal lattice fringes of 0.388 and 0.365 nm can be observed in Figure 2d. The resolved d spacing matched well with the (101) planes of TiO_2_.

**Figure 3:**
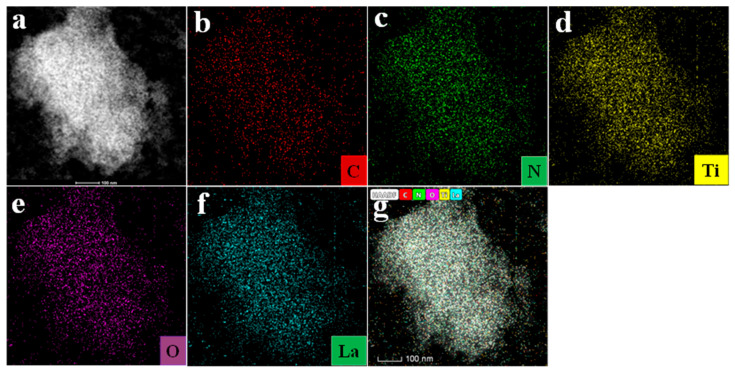
(**a**–**g**) TEM mapping images of CN/La-N-TiO_2_ photocatalyst at high magnifications.

The TEM elemental mapping images of CN/La-N-TiO_2_ are shown in Figure 3a–g, which reveal the uniform and homogenous presence of Ti, O, N, and La elements and further demonstrate that La-N-TiO_2_ nanoparticles were evenly dispersed over the g-C_3_N_4_ nanosheet. It may be beneficial for the interfacial electron transfer between g-C_3_N_4_/La-N-TiO_2_.

**Figure 4:**
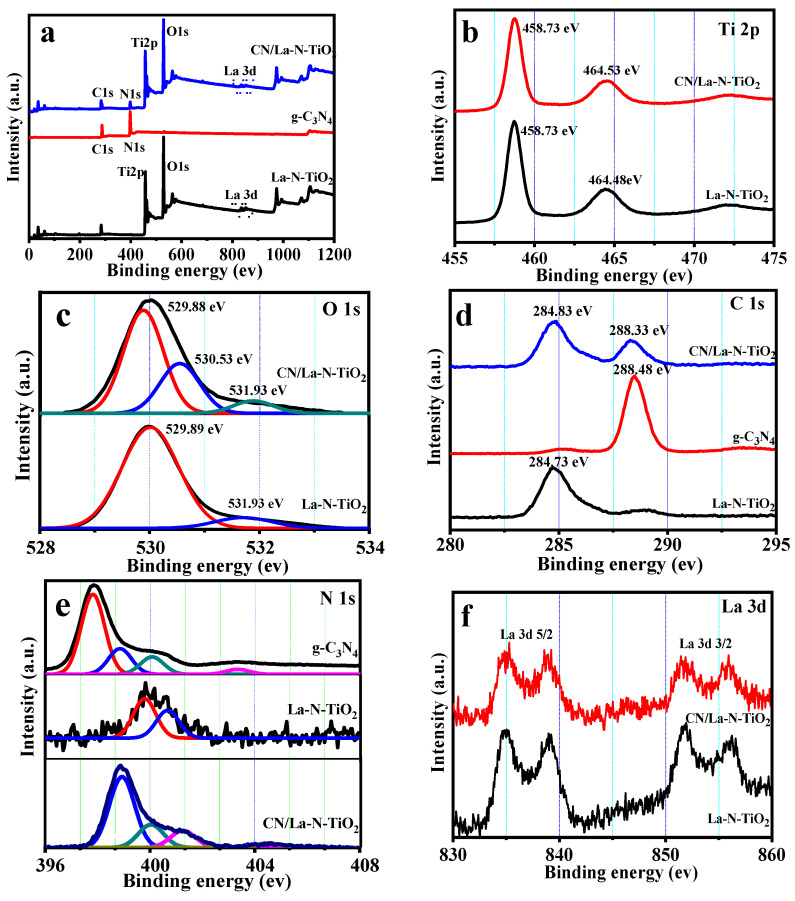
XPS survey spectrum (**a**); high-resolution XPS spectra of Ti 2p (**b**), O 1s (**c**), C 1s (**d**), N 1s (**e**), and La 3d (**f**).

The composition and element valence of g-C_3_N_4_, La-N-TiO_2_, and CN/La-N-TiO_2_ materials were investigated by XPS measurement (Figure 4). The full survey XPS spectrum (Figure 4a) shows obvious signals of Ti 2p, O 1s, C 1s, N 1s, and La 3d, clearly indicating the existence of C, O, Ti, N, and La in CN/La-N-TiO_2_. This result is consistent with the chemical composition, as proven by the XRD and TEM mapping.

#### 3.1.3. XPS Analysis

As can be seen from Figure 4b, binding energy peaks at 458.7 and 464.4 eV in the high-resolution Ti 2p spectrum are assigned to Ti 2p_3/2_ and Ti 2p_1/2_, respectively, revealing the characteristic value of Ti(IV) oxidation state in TiO_2_ [42]. Compared with La-N-TiO_2_, there is little change in Ti 2p peaks for the CN/La-N-TiO_2_. 

The high-resolution O 1s XPS spectrum (Figure 4c) of La-N-TiO_2_ is fitted with two peaks centered at 529.89 and 531.93 eV, which correspond to Ti-O-Ti and O-H species, respectively. Three fitted peaks located around 529.88, 530.53, and 531.93 eV in the high-resolution O 1s spectrum of CN/La-N-TiO_2_ correspond to Ti-O-Ti, O-H species, and adsorbed molecular oxygen groups, respectively [39]. There is a positive shift of O 1s peaks for the CN/La-N-TiO_2_ (from 529.88 to 529.89 eV). This shift is a consequence of the interaction between La-N-TiO_2_ and g-C_3_N_4_ in heterojunctions to increase the effective positive charge on the O species [27].

There are mainly two carbon species presented in the C1s spectra (Figure 4d), which are ascribed to the C-C and/or adventitious carbon at 284.83 eV and sp^2^-bonded carbon atom in the N-C=N group at 288.33 eV, respectively [23]. The sp^2^-bonded carbon atom is considered the major carbon in g-C_3_N_4_. 

The N 1s spectra of g-C_3_N_4_ could be fitted into three peaks (sp^2^-hybridized N involved in triazine rings (C-N=C) at 396.70 eV, tertiary N group (N-(C)_3_) at 398.70 eV and uncondensed amino group (-NH_x_) in the aromatic skeleton at 400.30 eV), indicating the presence of sp^2^-bonded graphitic carbon nitride (g-C_3_N_4_). The weak peak signal at 403.4 eV is attributed to the charge effect or positive charge localization of the heterocycles. The basic substructure units of g-C_3_N_4_ polymers [55]. There are two peaks in the XPS spectrum of N1s at 399.70 eV and 400.60 eV for La-N-TiO_2_, which suggest two coexisting chemical states of the N in doped TiO_2_ substitution doping (N-Ti–O) and N existed in the TiO_2_ lattice (Ti–O–N–O) [43]. The N 1s spectra of CN/La-N-TiO_2_ could be deconvoluted into four peaks at 398.5 eV, 398.8 eV, 400.1 eV, and 401.3 eV, respectively, corresponding to sp^2^-hybridized N (C-N=C), tertiary N group (N-(C)_3_), uncondensed amino group (-NH_x_), and substitution doping nitrogen (N-Ti–O). Compared with g-C_3_N_4_ and La-N-TiO_2_, there is an obvious binding energy shift of the N 1speaks in CN/La-N-TiO_2_, indicating that strong interaction exists between g-C_3_N_4_ and La-N-TiO_2_ [42,54].

According to the XPS investigation (as shown in Figure 4f), there are four peaks located at (834.8 eV, 838.40 eV) and (852.35 eV, 855.54 eV), which are attributed to La 3d_5/2_ and La 3d_3/2_, respectively, confirming that La^3+^ has been successfully doped into CN/La-N-TiO_2_ samples. As the radius of La^3+^(1.03 Å) is larger than the radius of Ti^4+^ (0.745 Å), La^3+^ can only form the La-O-Ti bond on the surface, and the La-O-La bond on the interstitial site of TiO_2_ lattice, instead of entering into the bulk lattice of TiO_2_ [41,57]. 

The atomic ratios of the relative elements, calculated from the XPS spectra, are summarized in Table 2. The C/N ratios for all cases of heating dicyandiamide are lower than 0.75 for the ideal crystal g-C_3_N_4_ [23,51,52,53,55]. From Table 2, the g-C_3_N_4_ nanosheet in this work is the ideal crystal. Combining the experimental results of XRD, SEM, TEM, and XPS, it can be concluded that the chemical combination existed on the interface between La-N-TiO_2_ and g-C_3_N_4_ nanosheet. 

#### 3.1.4. BET Surface Area Analysis

**Figure 5:**
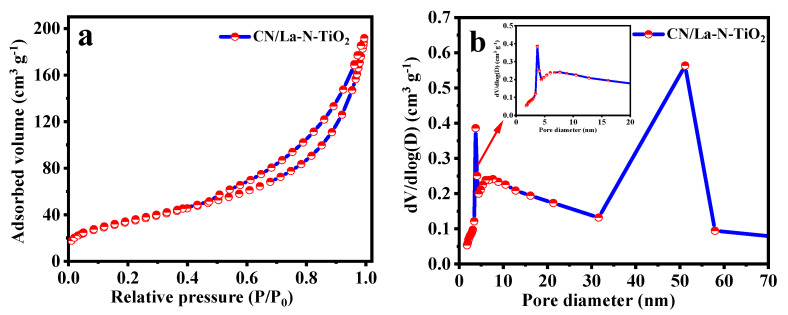
(**a**) N_2_ adsorption−–desorption isotherms and (**b**) the corresponding pore size distribution plots of CN/La-N-TiO_2_.

In Figure 5a, the N_2_ adsorption/desorption isotherm of CN/La-N-TiO_2_ shows a typical type IV curve with an H1-type hysteresis loop according to IUPAC classification, confirming the presence of mesopores [42]. Two peaks are observed from the pore distribution curve (Figure 5b), one at 4 nm is attributable to the confined self-assembled La-N-TiO_2_ component nanoparticles, and the other broad peak centering at 50 nm is probably formed from gaps between CN nanosheets and La-N-TiO_2_, caused by the steric confinement of crumpled and entangled nanosheet structure of seeded CN against La-N-TiO_2_ growth [56]. The data of S_BET_, total pore volume, and average pore diameter of La-N-TiO_2_, g-C_3_N_4_, and CN/La-N-TiO_2_ are summarized in Table 2. Compared with g-C_3_N_4_ (50 m^2^·g^−1^) and La-N-TiO_2_ (83 m^2^·g^−1^), CN/La-N-TiO_2_ possesses the highest surface area of ∼122 m^2^·g^−1^ and larger pore size of ∼8.76 nm and the total pore volume of 0.28 cm^3^·g^−1^. The higher specific surface areas of the as−prepared hybrids benefit the penetration and adsorption of pollutants on the surface of photocatalysts, which may result in a quick transport of reaction species onto the active sites.

#### 3.1.5. Spectral Properties

**Figure 6:**
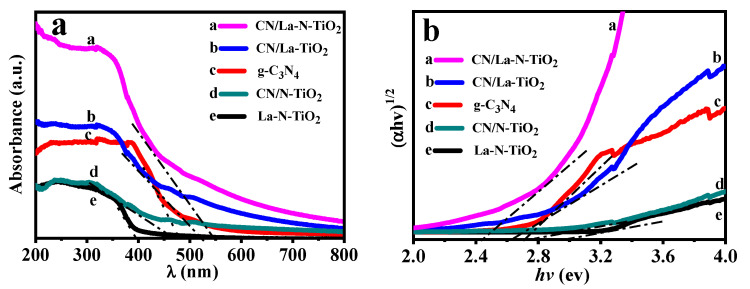
UV-vis spectra (**a**) of g-C_3_N_4_ La-N-TiO_2_, CN/N-TiO_2_, CN/La-TiO_2_, and CN/La-N-TiO_2_ composites, and (**b**) a plot of (αhυ)^1/2^ versus the photon energy (hυ).

The characteristic light absorption spectra of the catalysts are presented in Figure 6a. The absorption edges of La-N-TiO_2_ and g-C_3_N_4_ are 400 nm and 475 nm, respectively. The absorption edges of CN/La-TiO_2_ and CN/La-N-TiO_2_ redshift to 525 nm and 550 nm. In addition, their light absorption intensities further increased compared with g-C_3_N_4_ and La-N-TiO_2_, which indicates the broadened light absorption range and the enhanced light-harvesting capacity of CN/La-TiO_2_ and CN/La-N-TiO_2_. Compared with g-C_3_N_4_, CN/N-TiO_2_ shows a weakened light absorption intensity and a narrowed light absorption range, which may be due to the additional amount of g-C_3_N_4_. The bandgap energy (E_g_) is the important parameter for predicting photophysical and photochemical properties of semiconductors [42,58]. The E_g_ is determined by using the relationship between (αhυ)^1/2^ and photon energy and obtained through the linear extrapolation of the intercepts on the x axis (Figure 6b). The E_g_ of g-C_3_N_4_, La-N-TiO_2_, CN/N-TiO_2_, CN/La-TiO_2_, and CN/La-N-TiO_2_ are thereby estimated to be 2.70 eV, 2.98 eV, 2.77 eV, 2.62 eV, and 2.46 eV, respectively, which is consistent with the redshift of their absorption edges. 

### 3.2. Photocatalytic Performance

**Figure 7:**
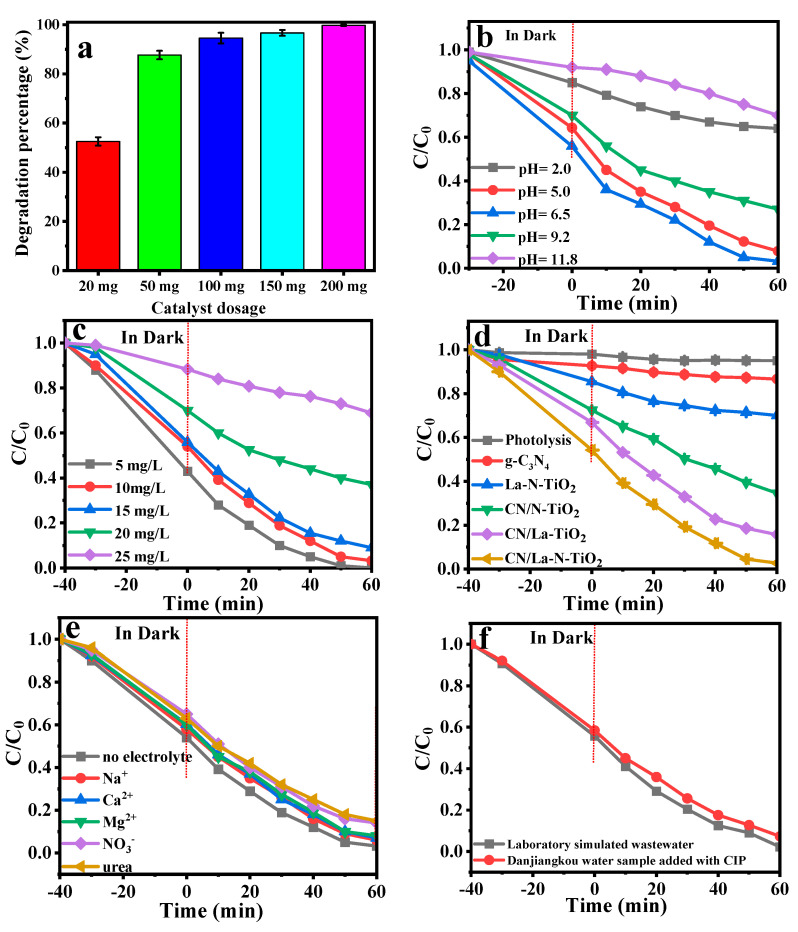
Photocatalytic degradation of ciprofloxacin under simulated solar- light irradiation (**a**) amount of catalyst, (**b**) at different initial pH, (**c**) Effects of the initial concentration of CIP (5–25 mg/L), (**d**) by different photocatalysts, (**e**) Effect of various electrolytes, and (**f**) in actual water samples in the presence of CN/La-N-TiO_2_.

Many studies have demonstrated that catalyst dosage, solution pH, and coexisting cations have a considerable impact on the removal efficiency of the pollutant. From an economic perspective and for designing large-scale treatment processes, the determination of optimum parameters for the removal of CIP by CN/La-N-TiO_2_ is important. The main influential factors such as pH of the solution, initial CIP concentration, catalyst dosage, and -coexisting ions were investigated in detail and shown in Figure 7a–f.

#### 3.2.1. Effect of Catalyst Dosage

The effect of catalyst dosage was explored and shown in Figure 7a. When the CN/La-N-TiO_2_ dosage increased from 0.1 g/L to 1 g/L, the removal efficiency of CIP increased from 51.5% to 96.8%. This is because the number of the active sites on the catalyst surface for radical formation increased by increasing the catalyst dosage, leading to a higher photocatalysis activity. However, when the CN/La-N-TiO_2_ dosage was further increased above 0.75 g/L, the removal efficiency of CIP did not significantly vary. For the above-mentioned facts, 150 mg was considered the optimum dosage for the treatment of 200 mL CIP solution with a concentration of 10 mg/L.

#### 3.2.2. Effect of pH

**Figure 8:**
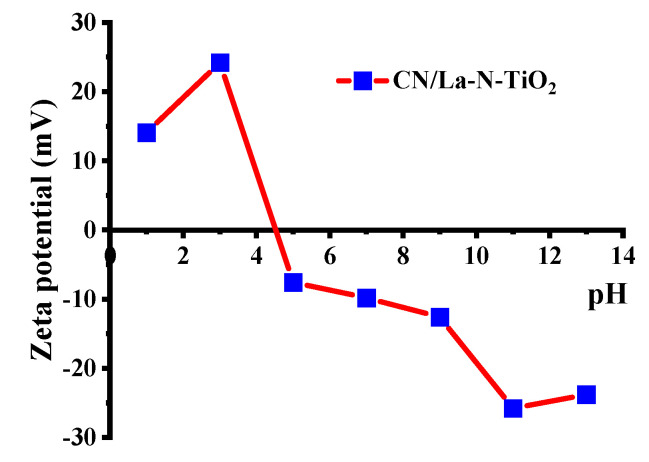
Zeta potential of CN/La-N-TiO_2_ in different pH conditions.

In Figure 8, the zeta potential values measured for CN/La-N-TiO_2_ as a function of pH are plotted. It is observed that at pH values around 3 the zeta potential is about +24 mV, but at pH values between 4 and 5, there is a rapid decrease of the zeta potential, the IEP occurring at pH 4.6 [59]. Hence, the catalyst surface is negatively charged at above a value of pH 4.6, while it is positively charged at below a value of pH 4.6. CIP is an amphoteric drug with a p*K*_a_ value of 6.1 (for –COOH) and 8.9 (for -NH- and -N- on the piperazinyl ring), where the isoelectric point lies at pH = 7.4 [28,60]. Therefore, CIP zwitterion will present three different ionic forms, mainly depending on the pH value of the solution. As CIP and CN/La-N-TiO_2_ are pH sensitive, pH will influence the characteristics of both CIP and CN/La-N-TiO_2_ as well as the interactions between them which change as pH changes.

The plots of the effect of pH (2–11.8) on CIP photodegradation with an initial concentration of 10 mg L^−1^ were studied in presence of CN/La-N-TiO_2_ under visible light and shown in Figure 7b. The 0.05 mol·L^−1^ HCl or 0.05 mol·L^−1^ NaOH solution was used to adjust the pH value of the simulated CIP wastewater solution. It can be seen from Figure 7b, the degradation is strongly inhibited at highly acidic and basic pH and the degradation efficiency reduces from 96.8% (pH = 6.5) to 35.2% at pH = 2 and 30.5% at pH = 11.8. It is because CIP and CN/La-N-TiO_2_ are negatively charged leading to decreased interactions at higher pH, while the generation of ·OH radicals is inhibited in an alkaline medium. It can be concluded that the optimum pH value of the CIP wastewater solution is in the pH range 6–7.

#### 3.2.3. Effect of Initial CIP Concentrations

It can be found in Figure 7c that CIP with an initial concentration of 5 mg/L has been completely removed after 60 min of irradiation, while the relatively stable degradation efficiency is observed when the concentration increases to 10 mg/L. However, a further increase in initial CIP concentration from 10 to 20 mg/L resulted in a decrease in degradation efficiency from 96.8% to 31.2%. This is probably due to the number of surface-active sites on the catalyst provided by 150 mg CN/La-N-TiO_2_ are not enough for a higher initial CIP concentration. Additionally, the higher the concentration, the more intermediates are generated. The generated intermediates would compete with the CIP molecules to contact the active sites on the catalyst surface, so as to inhibit the degradation of CIP. 

#### 3.2.4. Different Photocatalysts

Under the optimal conditions, where the pH value is 6.5, initial CIP concentration is 10 mg·L^−1^, and the catalyst dosage is 0.75 g/L, the degradation experiment of CIP was carried out. As shown in Figure 7d, on a blank experiment, the self-photolysis removal of CIP was negligible. Under simulated solar- light irradiation, 15.2 %, 30.2%, 65.4%, 85.8%, and 96.8% of the CIP were degraded in 60 min by g-C_3_N_4_, La-N-TiO_2_, CN/N-TiO_2_, CN/La-TiO_2_, and CN/La-N-TiO_2_, respectively. CN/La-N-TiO_2_ shows the highest CIP degradation of 96.8% in 60 min of irradiation followed by CN/La-TiO_2_ (85.8%) and CN/N-TiO_2_ (65.4%), indicating that the combination among La-N-co-doped TiO_2_ and g-C_3_N_4_ hybrid photocatalyst can significantly enhance the photocatalytic properties. 

#### 3.2.5. Effect of Coexisting Cations

Natural water body contains various ions and organics, which would affect the photodegradation of CIP. In this work, removal of CIP by CN/La-N-TiO_2_ was tested in presence of Na^+^ (10 mg·L^−1^), Ca^2+^ (10 mg·L^−1^), Mg^2+^ (10 mg·L^−1^), NO_3_^−^ (10 mg·L^−1^), and urea (5 mg·L^−1^) (Figure 7e). The concentrations of ions and urea used are representative of Danjiangkou’s natural water body. It can be found in Figure 7e that Na^+^, Ca^2+^, and Mg^2+^ have little effect on photocatalytic activity, where NO_3_^−^ and urea obviously hindered the photocatalytic degradation of CIP.

#### 3.2.6. Degradation in Water Sample of Dangjiangkou

In order to study the effect of the actual water application of CN/La-N-TiO_2_. The degradation experiment of CIP in the water sample of Dangjiangkou was studied. As shown in Figure 7f, there is little reduction in degradation rate compared to laboratory simulated CIP wastewater. Therefore, CN/La-N-TiO_2_ has a good practical application prospect.

**Figure 9:**
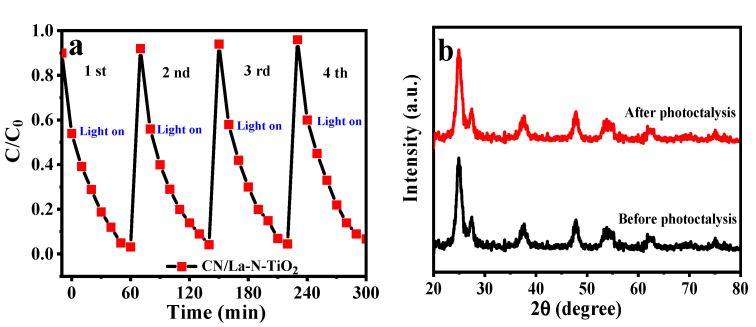
(**a**) Cycling experiment for the CIP removal over CN/La-N-TiO_2_ and (**b**) XRD patterns of CN/La-N-TiO_2_ before and after the cycling experiment.

Furthermore, the stability and reusability of CN/La-N-TiO_2_ were evaluated via four successive cycles of CIP photocatalytic degradation experiments, where the removal rate of CIP remained almost unchanged in the cycles (Figure 9a). Meanwhile, to further explore the photocatalytic stability, the XRD patterns of CN/La-N-TiO_2_ before and after the cycling experiment were examined (Figure 9b). It can be found that the crystallinity was almost unchanged throughout the 4 cycles. Thus, the synthesized CN/La-N-TiO_2_ exhibits good stability and reusability, a property that is very in demand for practical applications.

#### 3.2.7. Proposed Pathways of CIP Degradation

**Figure 10:**
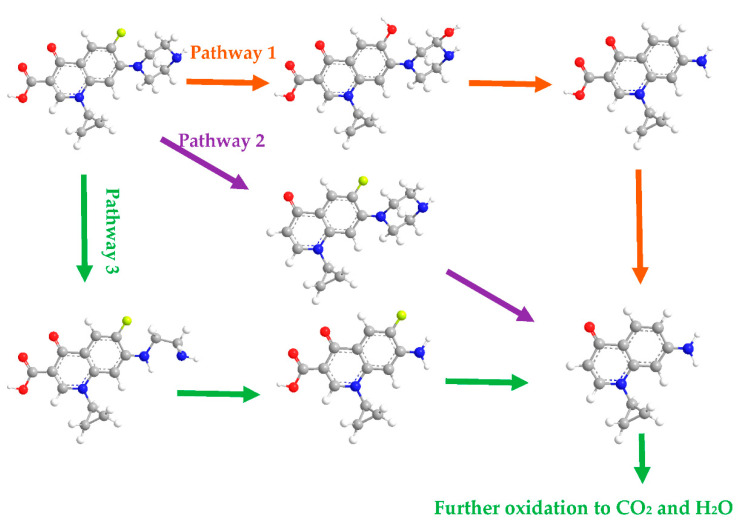
Suggested pathways for ciprofloxacin degradation in CN/La-N-TiO_2_.

In order to estimate the toxicity of intermediates and better elucidate the mineralization pathway of CIP, the byproducts formed during the 10 min and 30 min -photocatalytic experiment were analyzed by HR-MS. According to the byproducts and a literature study [9,10,12,17,19,61], the probable degradation pathway is conceived and proposed in Figure 10. A high-intensity peak (*m*/*z* = 332) is corresponding with the original CIP molecule and the intensity of the CIP peak decreased to 20% after 10 min of degradation reaction in presence of CN/La-N-TiO_2_ under visible light. Beyond CIP, six other -byproducts are detected including BP-1 (*m/z* = 362), BP-5 (*m/z* = 346), BP-2 (*m/z* = 306), BP-3 (*m/z* = 263), BP-4 (*m/z* = 246), and BP-6 (*m/z* = 200) (In Figure S2). It can be observed that ··O_2_^−^and h^+^ direct attack as the main attacking species to attack the aromatic tertiary amine group, carboxyl group, and the secondary aliphatic amine group in the piperazine ring, which are three principal reactive sites in CIP in this photocatalytic system [62]. Generally, the intermediates formed showed less toxicity than the original chemical, which proved that CN/La-N-TiO_2_ is a promising photocatalyst in the CIP wastewater treatment [10,12].

#### 3.2.8. Radical Trapping Experiment and Possible Photocatalytic Mechanism

**Scheme 1:**
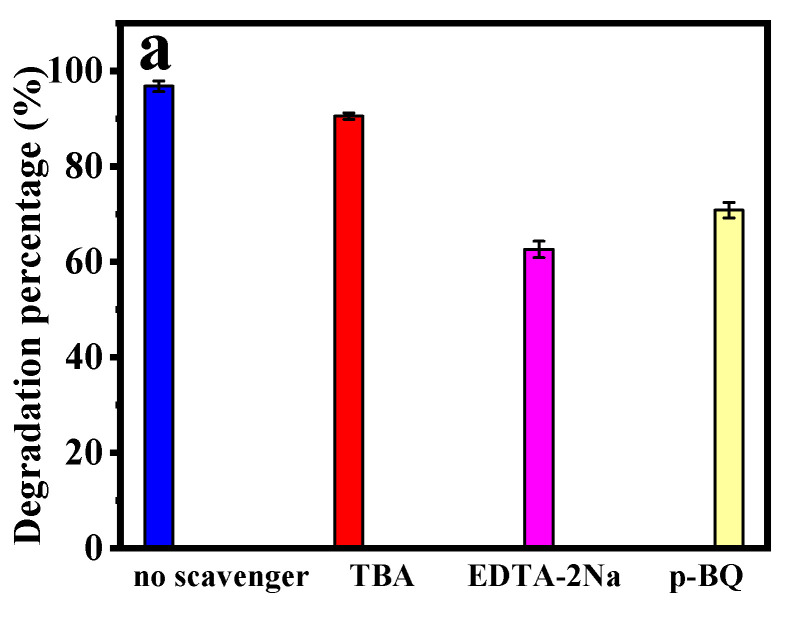

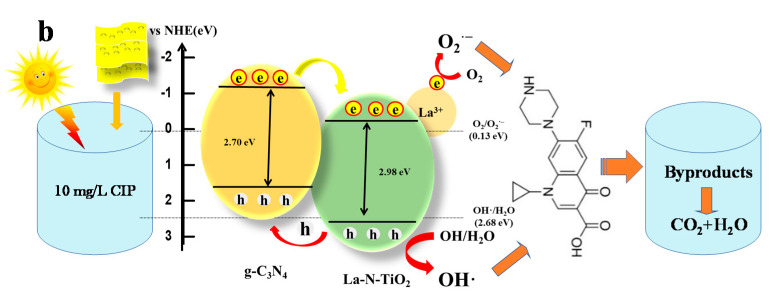
(**a**) Photocatalytic activity of CN/La-N-TiO_2_ toward CIP degradation in the presence of different scavengers and (**b**) schematic diagram for photocatalytic removal CIP mechanism by CN/La-N-TiO_2_ photocatalyst under simulated solar light irradiation.

In order to identify the active oxidative species in the photocatalytic process, trapping experiments are carried out in the CN/La-N-TiO_2_ hybrid photocatalytic system. Scheme 1a shows the photocatalytic degradation rate of CN/La-N-TiO_2_ in the presence of different scavengers. The photocatalytic efficiency decreases a little by the addition of t-BuOH (TBA, ·OH scavenger, decreases to 90.5%), while it is remarkably suppressed when EDTA-2Na (h^+^ scavenger decreases to 62.6%) and p-Benzoquinone (p-BQ, ·O_2_^−^ decreases to 70.8%) were added to the system. Therefore, it can be inferred that the active species affect the photocatalytic activity in the following order: h^+^ > ·O_2_^−^ > ·OH [32]. 

Based on the above results, here a possible photocatalytic mechanism of g-C_3_N_4_/La-N-TiO_2_ composite was discussed and shown in Scheme 1b. The following empirical equation: $E_{VB}=\chi -E_{c}+0.5E_{g}$ were also employed to calculate the VB and CB potentials, where χ is the electronegativity of the semiconductor. For g-C_3_N_4_ and TiO_2_, χ is 4.73 and 5.81, respectively [42,45]. E_c_ is the energy of free electrons based on the hydrogen scale (4.50 eV). The E_VB_ and E_CB_ edge potentials of La-N-TiO_2_ were calculated to be +2.80 eV and −0.18 eV, respectively, and those of g-C_3_N_4_ were +1.55 eV and −1.25 eV, respectively. 

Under simulated solar light illumination, both g-C_3_N_4_ and La-N-TiO_2_ could be stimulated and generate electron-hole (e^−^-h^+^) pairs [42,45]. Due to the CB potential of g-C_3_N_4_ (−1.25 eV) being lower than that of La-N-TiO_2_ (−0.18 eV), the photo-induced electrons would shift from the CB of g-C_3_N_4_ to the CB of g-C_3_N_4_ through the heterointerface. The e^−^ generated in g-C_3_N_4_ goes to TiO_2_, where La^3+^ with 4f orbit can easily capture O_2_ adsorbed on the surface of TiO_2_ to form superoxide anion (·O_2_^−^) [23,41,45,56].

Part of the holes (h^+^) in the VB of TiO_2_ can easily be transferred to the VB of g-C_3_N_4_. Due to the lower reduction potential of the VB of g-C_3_N_4_ (1.55 eV vs. NHE), h^+^ in the VB of g-C_3_N_4_ can be used to degrade CIP directly. The remaining h^+^ at the VB potential of La-N-TiO_2_ (2.80 V vs. NHE) can oxidize water into hydroxyl radicals (·OH), owing to their potential being higher than the H_2_O/·OH potential (2.68 eV vs. NHE) [42]. Thus, during the photocatalytic reaction, h + and ·O_2_^−^ are the most important active species.

(1) $$g-C_{3}N_{4}+\mathrm{hv}\to \;g-C_{3}N_{4}\;\left(e^{-}+h^{+}\right)$$

(2) $$\mathrm{La}-N-{\mathrm{TiO}}_{2}+\mathrm{hv}\;\to \;\mathrm{La}-N-{\mathrm{TiO}}_{2}\;\left(e^{-}+h^{+}\right)$$

(3) $$h^{+}+H_{2}O\;\to \;\cdot \mathrm{OH}$$

(4) $${\mathrm{La}}^{3+}+e^{-}+O_{2}\to \;\cdot O_{2}{}^{-}$$

(5) $$h^{+}\left(\mathrm{main}\right)+\cdot O_{2}{}^{-}\left(\mathrm{main}\right)+\cdot \mathrm{OH}+\mathrm{CIP}\to \mathrm{degradation}\;\mathrm{products}$$

## 4. Conclusions

A 3D nanocomposite photocatalyst g-C_3_N_4_/La-N-TiO_2_ was successfully synthesized by a simple and reproducible in-situ synthetic method. g-C_3_N_4_/La-N-TiO_2_ exhibits high photocatalytic activity for the degradation of ciprofloxacin. A 96.8% degradation of CIP was achieved in 60 min under the conditions of 0.75 g·L^−1^ CN/La-N-TiO_2_, 10 mg·L^−1^ CIP, and pH of 6.5. The excellent photocatalytic performance of CN/La-N-TiO_2_ on degrading CIF is attributed to the formation of a junction that efficiently improves charge separation and wide spectrum response. The removal rates were rarely affected by Na^+^ (10 mg·L^−1^), Ca^2+^ (10 mg·L^−1^), Mg^2+^ (10 mg·L^−1^), and urea (5 mg·L^−1^), where the concentrations of ions and urea used are representative of Danjiangkou natural water body. In addition, the intermediates formed showed less toxicity than the original chemical, which proved that CN/La-N-TiO_2_ nanocomposite is a green and promising photocatalyst for large-scale applications and would be a candidate for the removal of the emerging antibiotics present in the water environment.

## Figures and Tables

**Table 1:** Photocatalytic activity of semiconductor materials for CIP degradation under visible light irradiation.

Photocatalysts	C_0_ (mg/L)	pH	Degradation Rate (%)	Light Irradiation Wavelength (λ)	Incident Light Power	Ref
Commercial anatase TiO_2_	1500 ppm	5.8	Kapp = 0.02 min^−1^	Simulated visible light	Commercial visible metal halide lamp	[24]
CeO_2_–Ag/AgBr	10	-	93.05%/120 min	λ > 420 nm	A 300 W Xe lamp	[25]
FeS/Fe_2_S_3_/zeolite	2	-	6%/90 min	λ > 380 nm	Tungsten lamp	[26]
Mesoporous TiO_2_ nanocrystallite aggregates	160	-	96.05%/6 h	200 nm ~1000 nm	A 500 W Xe lamp	[27]
ZnO	4	7/10	48%/60 min	365 nm	A xenon lamp	[28]
3D γ-Fe_2_O_3_@ZnO core-shell photocatalyst	10	5.8	92.5%/60 min	Simulated sunlight	A 300 W Xenon lamp	[29]
Bi_2_O_3_/(BiO)_2_CO_3_	10	4.0~8.3	93.4%/30 min	Simulated solar light	A 300 W Xenon lamp	[30]
Ag@PCNS/BiVO_4_	10	-	92.6%/120 min 17.5%/120 min	λ > 420 nm λ > 760 nm	-	[31]
ZnAl-LDH/g-C_3_N_4_	20	-	84.1%/150 min	-	A 35 W Xenon lamp	[35]
Ag_2_CrO_4_/Ag/BiFeO_3_@RGO	10	6	96%/60 min	Visible light	450 mW cm^−2^	[33]
TiO_2_ nanorod/g-C_3_N_4_ nanosheet	4.97	6.3	93.4%/60 min	Simulated sunlight	A 500WXenon lamp	[34]
TiO_2_@g-C_3_N_4_ hollow	-	-	74%/120 min	Simulated sunlight	-	[32]
CN/La-N-TiO_2_	10	6.0–7.0	96.8%/60 min	λ > 420 nm	A 300 W Xe lamp	our work

C_0_: the initial concentration. PMS: peroxymonosulfate.

**Table 2:** Atom concentrations, specific surface area (S_BET_), pore size, and band gap of the synthesized samples.

Catalysts	C_1_/at.%	C_2_/at.%	Ti/at.%	O/at.%	N/at.%	La/at.%	Surface Area (m^2^/g)	Av Pore Size(nm)	Pore Volume (cm^3^/g)
La-N-TiO_2_	15.71	–	19.51	51.62	1.55	0.6	83 [47]	5.10	0.17
g-C_3_N_4_	–	31.298	–	–	66.789	–	50 [42]	–	–
CN/La-N-TiO_2_	12.64	6.94	19.15	48.69	12.52	0.06	122	8.76	0.28

## Supplementary Materials

The following supporting information can be downloaded at: https://www.mdpi.com/article/10.3390/ijerph19084793/s1, Figure S1: (a) XRD pattern and (b) Photocatalytic degradation of CIP of the composites of CN/La-N-TiO_2_ composite with with different amount of dicyanide. Figure S2: Mass spectrometry of by-products for ciprofloxacin degradation in CN/La-N-TiO_2_.
