# Supplementary material for: High Photocatalytic Activity of g-C3N4/La-N-TiO2 Composite with Nanoscale Heterojunctions for Degradation of Ciprofloxacin
Source: Int J Environ Res Public Health. 2022 Apr 15;19(8):4793. doi: 10.3390/ijerph19084793 (PMC9027728; doi:10.3390/ijerph19084793)
Supplement: Supplementary file 1 [file ijerph-19-04793-s001.zip › ijerph-1642363-supplementary.pdf]

High photocatalytic activity of g-C_3N_4/La-N-TiO_2 composite with nanoscale heterojunctions for degradation of ciprofloxacin

Yanmin Yu [1], Ke Liu [2], Yangyang Zhang [1], Xuan Xing [3,*] and Hua Li [3,*]

[1] School of Basic Medicine, Hubei University of Arts and Science, Xiangyang 441053, China; yuyanmin2005@163.com (Y.Y.); yangyangzhang2017@163.com (Y.Z.)
[2] Hubei Key Laboratory of Low Dimensional Optoelectronic Materials and Devices, Hubei University of Arts and Science, Xiangyang 441053, China; liuke@hbuas.edu.cn
[3] College of Life and Environmental Sciences, Minzu University of China, Beijing 100081, China
* Correspondence: Xingxuanpku@163.com (X.X.); lihua@muc.edu.cn (H.L.)

Figure S1

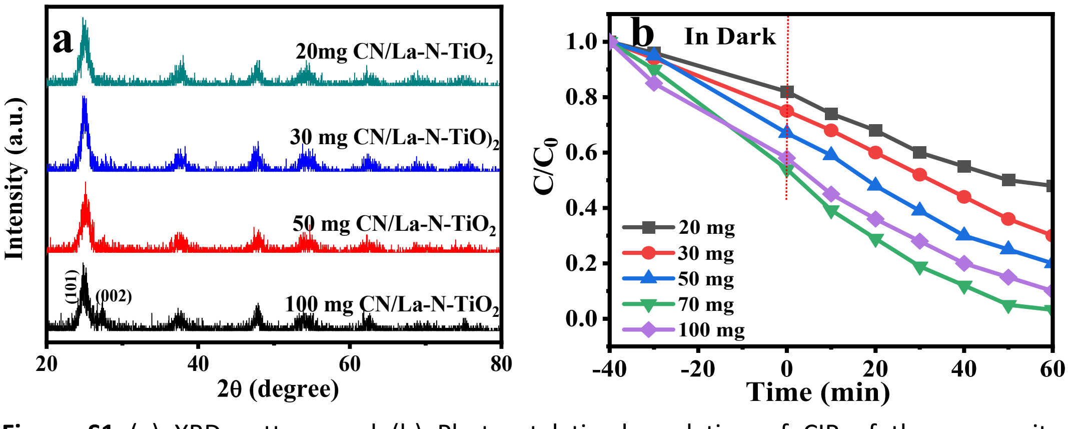

Figure S1 (a) XRD pattern and (b) Photocatalytic degradation of CIP of the composites of CN/La-N-TiO_2 composite with with different amount of dicyanide.

Figure S2

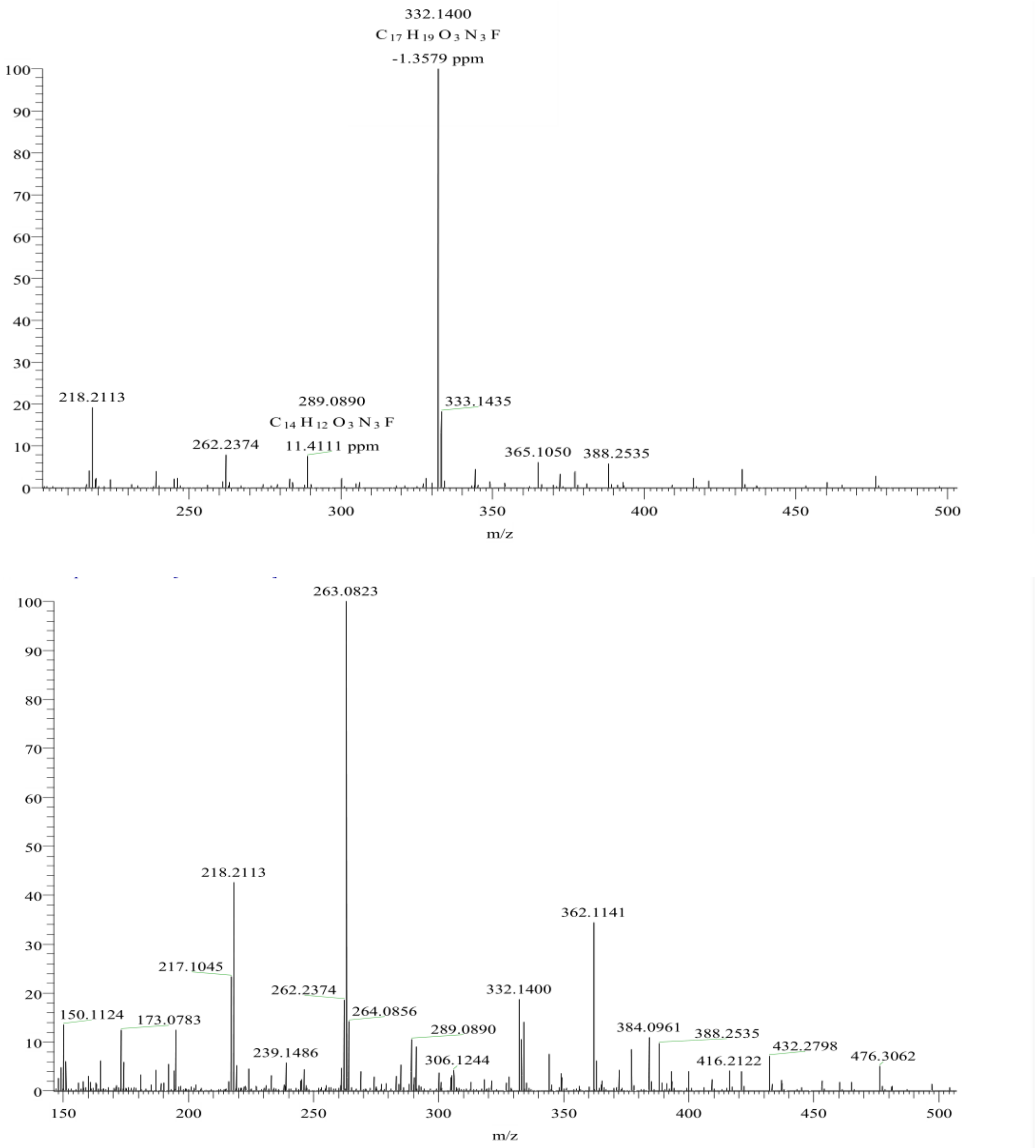

Figure S2 Mass spectrometry of by-products for ciprofloxacin degradation in CN/La-N-TiO_2.
